# Edition of TFAM gene by CRISPR/Cas9 technology in bovine model

**DOI:** 10.1371/journal.pone.0213376

**Published:** 2019-03-07

**Authors:** Vanessa Cristina de Oliveira, Gabriel Sassarão Alves Moreira, Fabiana Fernandes Bressan, Clésio Gomes Mariano Junior, Kelly Cristine Santos Roballo, Marine Charpentier, Jean-Paul Concordet, Flávio Vieira Meirelles, Carlos Eduardo Ambrósio

**Affiliations:** 1 Department of Veterinary Medicine, Faculty of Animal Science and Food Engineering, University of São Paulo, Pirassununga, São Paulo, Brazil; 2 Laboratoire Structure et Instabilité des Génomes, Museum National d’Histoire Naturelle, INSERM U1154, CNRS UMR7196, Paris, France; West China Hospital, Sichuan University, CHINA

## Abstract

The mitochondrial transcription factor A (*TFAM*) is a mitochondrial DNA (mtDNA) binding protein essential for the initiation of transcription and genome maintenance. Recently it was demonstrated that the primary role of *TFAM* is to maintain the integrity of mtDNA and that it is a key regulator of mtDNA copy number. It was also shown that TFAM plays a central role in the mtDNA stress-mediated inflammatory response. In our study, we proposed to evaluate the possibility of editing the *TFAM* gene by CRISPR/Cas9 technology in bovine fibroblasts, as *TFAM* regulates the replication specificity of mtDNA. We further attempted to maintain these cells in culture post edition in a medium supplemented with uridine and pyruvate to mimic Rho zero cells that are capable of surviving without mtDNA, because it is known that the *TFAM* gene is lethal in knockout mice and chicken. Moreover, we evaluated the effects of *TFAM* modification on mtDNA copy number. The CRISPR gRNA was designed to target exon 1 of the bovine *TFAM* gene and subsequently cloned. Fibroblasts were transfected with Cas9 and control plasmids. After 24 h of transfection, cells were analyzed by flow cytometry to evaluate the efficiency of transfection. The site directed-mutation frequency was assessed by T7 endonuclease assay, and cell clones were analyzed for mtDNA copy number by Sanger DNA sequencing. We achieved transfection efficiency of 51.3%. We selected 23 successfully transformed clones for further analysis, and seven of these exhibited directed mutations at the CRISPR/Cas9 targeted site. Moreover, we also found a decrease in mtDNA copy number in the gene edited clones compared to that in the controls. These *TFAM* gene mutant cells were viable in culture when supplemented with uridine and pyruvate. We conclude that this CRISPR/Cas9 design was efficient, resulting in seven heterozygous mutant clones and opening up the possibility to use these mutant cell lines as a model system to elucidate the role of *TFAM* in the maintenance of mtDNA integrity.

## Introduction

The mitochondrial transcription factor A (*TFAM*) is a member of the High Mobility Group Box (HMGB) subfamily structurally composed of 2 HMGB domains, HMG1 and HMG2, which binds to mtDNA promoters [1–4]. The *TFAM* gene plays an important role in cellular physiology involved in the maintenance of mtDNA, and regulates the number of mtDNA copies. It is also essential for the initiation of transcription of mtDNA genes [5–8].

*TFAM* is a candidate gene for investigation of its functions in transcription and replication of mitochondrial DNA [1, 9]. TFAM is required to regulate the number of copies of mtDNA [10–11] and is essential for embryonic development in mice [12]. In bovine oocytes at different embryonic stages, the great importance of TFAM in the maintenance of the first stages of embryogenesis has been reported [13].

The possibility of modifying cellular genome sequences has recently become a reality due to various gene-editing techniques, and this has many important applications, such as investigating the role of mutations in predisposition to diseases. Recently, gene-editing tools have been based on the CRISPR/Cas9 system (Clustered Regularly Interspaced Short Palindromic Repeats). This originates as a system found in bacteria and archaea and is an adaptive defense mechanism protecting against invasion of exogenous DNA [14].

CRISPR/Cas9 uses a short gRNA containing 20 nucleotides complementary to a DNA sequence, as well as an RNA-guided Cas9 nuclease. When gRNA binds to the target site, the Cas9 protein induces breaks in the two strands of DNA. CRISPR/Cas9 has emerged as a powerful tool that has been used in various applications, including human and veterinary medicine.

Regarded as a key protein in the mtDNA maintenance, the TFAM gene was silenced in mice by Cre-loxP technique, resulting in loss of embrionary lethality and mtDNA decrease. That research also showed the importance of TFAM in the molecular process involved in maintaining mtDNA integrity, allowing future development of works regarding this gene. Hence, our study proposed to edit the TFAM gene in bovine fibroblasts by CRISPR/Cas9 technology, firstly in order to assess if it’s possible to edit it by this tool, maintain the edited cells in culture since the embryonic TFAM disruption is lethal, and in the future characterize the generated cells and discover their application potential. Among genes that regulate mitochondrial transcription and replication activity, *TFAM* exerts considerable interspecific variability. In this context, we intended to edit the *TFAM* gene precisely because of the possibility of this gene being the main specificity regulator of mtDNA replication, allowing in the future to modify the TFAM origin and eventually control and repopulate a cell with its specific mitochondria harvested in different species.

## Materials and methods

Our study protocol was approved by the Research Ethics Committee (Approval No. 5828250215) of the Faculty of Animal Science and Food Engineering, University of São Paulo, Brazil.

### Cell line

Bovine fibroblasts used in this study were derived from a skin biopsy; the tissue was minced into small pieces and digested with collagenase type IV (Sigma C2674) for 3h at 37°C. The tissue was then centrifuged at 1500 rpm and the resultant pellet resuspended in Iscove’s modified Dulbecco’s medium (IMDM) (Gibco) supplemented with 10% fetal bovine serum (Hyclone) and antibiotics (5% penicillin–streptomycin; Invitrogen, Carlsbad, CA, USA).

### CRISPR

#### Design

**Fig 1:**
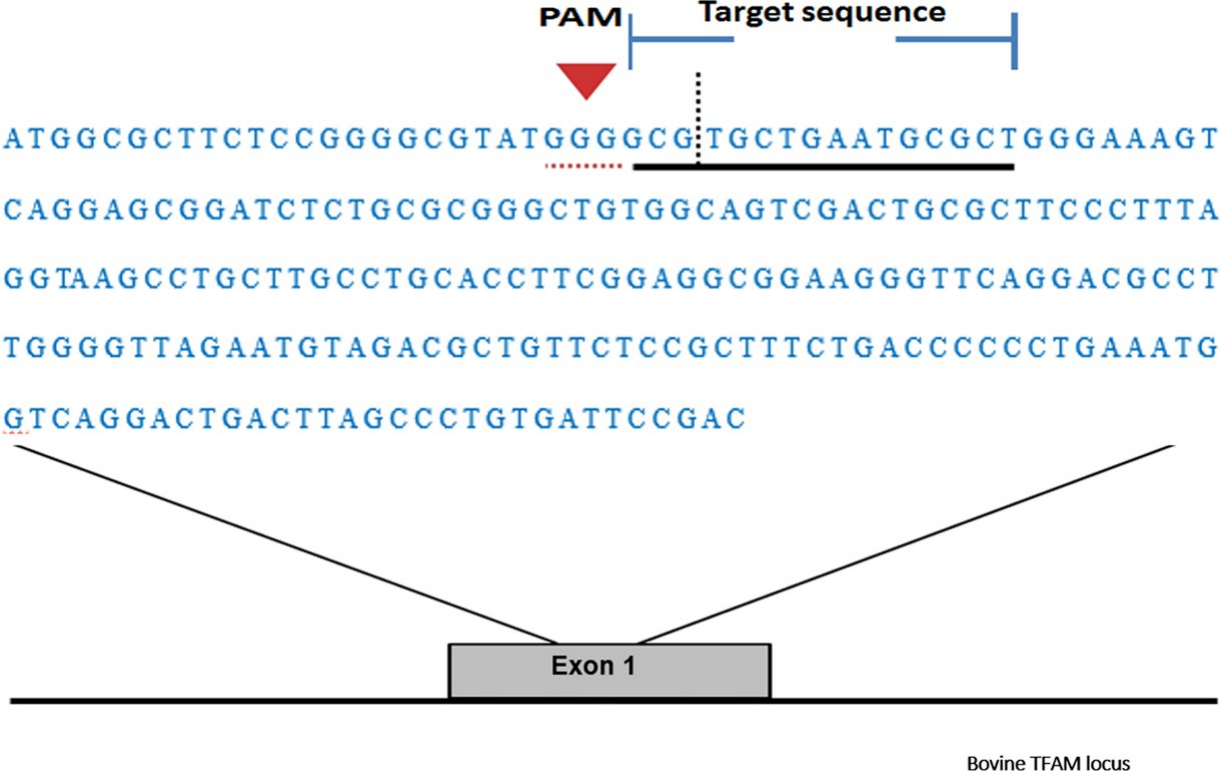
Scheme of guide RNA and PAM sequence targeting exon 1 in the bovine TFAM gene. Note the complete sequence of Exon 1. The horizontal red underline represents the PAM sequence. The horizontal black underlined region represents the guide sequence and the cut site is the vertical black dotted line.

The sequence of the *TFAM* gene was obtained from the GenBank NR database (www.ncbi.nlm.nih.gov). For CRISPR targeting, exon 1 DNA sequence of the bovine *TFAM* gene was entered into the ‘CRISPR Direct’ site (crispr.dbcls.jp) and also to the rgenome site (rgenome.net) so as to design the gRNA (Fig 1). The design of gRNA was facilitated using these freely available, online tools. We employed these tools to identify guide sequences and to minimize identical genomic matches or mismatches to reduce the risk of off-target modifications. We designed four gRNAs for evaluation.

#### Cloning-Hybridization of gRNA oligonucleotides

Oligos with gRNA sequences were resuspended at a concentration of 100μM in ddH_2_O and 5μL each of the sense and antisense primers were added to a mix of 35μL water, 5μL NEB2 buffer and hybridized at 90°C for 5 minutes, then cooled at room temperature for 2 hours.

#### Ligation reaction for annealed oligos

From the products of hybridization, 1μL was mixed with 5μL of T4 DNA ligase (Invitrogen) and 9μL of the vector mix containing linearized plasmid pMLM3636 (Addgene #43860), T4 Buffer, and water. The total volume reaction was 15μL. The mixture was incubated at 16°C for 1 hour and then at 65°C for 10 minutes to inactivate the enzyme.

### Transformation

Bacteria (*E*. *coli* C3019, NEB 10-beta) were transformed by heat shock as follows: 2μL of ligation reaction was mixed with 7μL bacteria, incubated on ice for 30 minutes, and then transferred to a water bath at 42°C for 30 seconds. They were added to 200μL of Luria-Bertani broth (LB) medium. The culture was then incubated at 37°C for 1 hour and subsequently spread on LB/Amp/Agar plates. The plates were incubated at 37°C for 16 hours until the appearance of colonies. Individual colonies were placed in 25mL of LB medium with ampicillin. These were incubated with constant agitation for 12 hours after which they were removed, subjected to DNA miniprep extraction (QIAprep Spin Miniprep Kit- cat. nos. 27104 and 27106), and followed by DNA sequencing.

### Transfection of bovine fibroblasts

**Fig 2:**
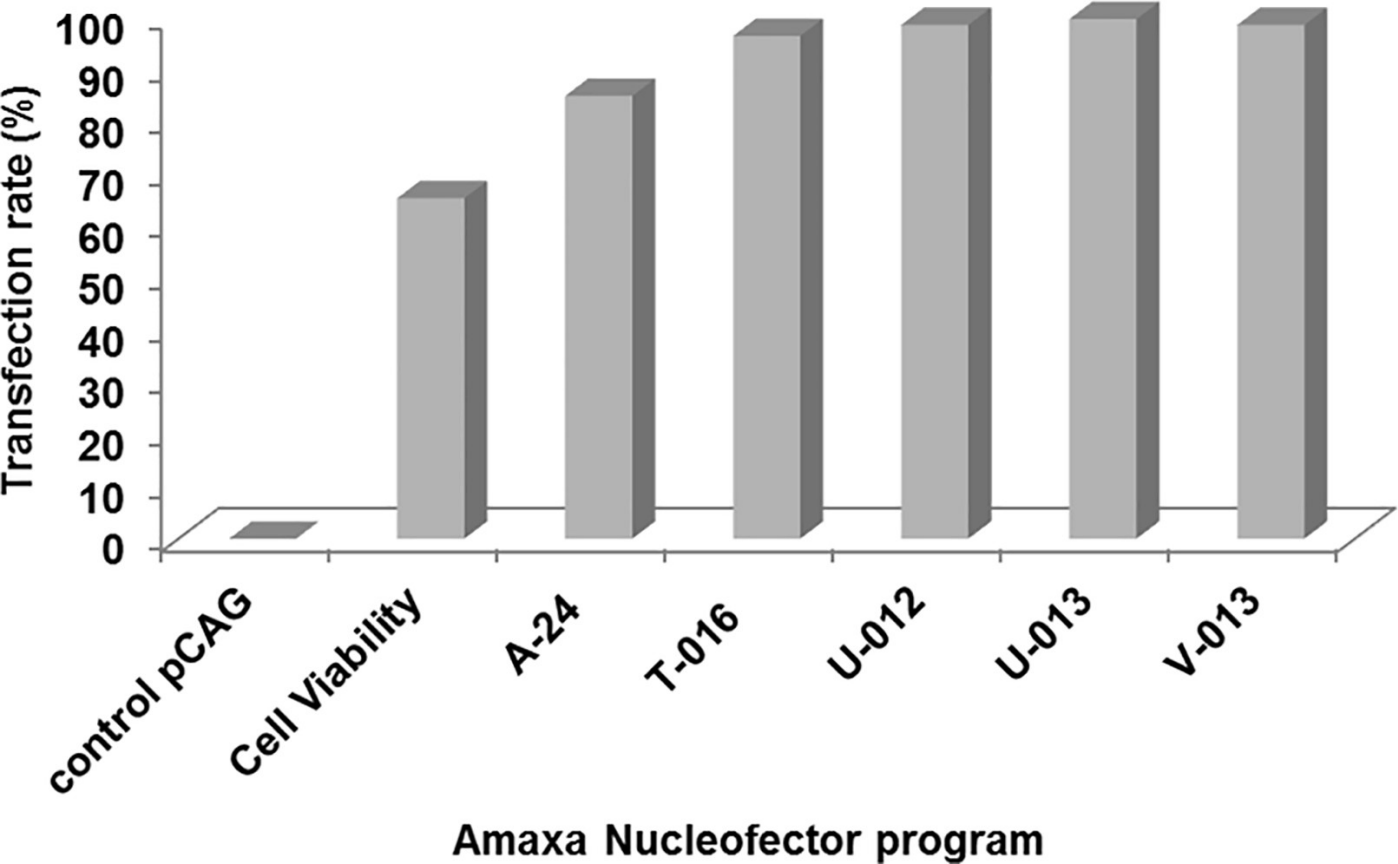
Test of Amaxa Nucleofector program by flow cytometry analysis. Control pCAG (0.0%), Cell viability (65.2%), A-24 program (84.8%), T-016 program (96.3%), U-012 (98.4%), U-013 (99.5%) and V-013 (98.4%).

Bovine fibroblasts cultured to 85% confluency were separated for transfection. Test assays were run to determine the most suitable program of Amaxa Nucleofector equipment. Control cells were transfected were transfected with pCAG expression vector (a kind gift of M. Jasin, similar to Addgene #26477 but lacking ISceI cDNA). We observed that all the programs tested (A-24, T-016, U-012, U-013 e V-013) led to a high rate of transfection (Fig 2). U-012 was chosen due to its high efficiency, resulting in 98.4% cell transfection and good cell viability after re-culturing.

After determining the most convenient program, we proceeded to transfection with Cas9 (Addgene 48668), with the same plasmids and we tested 4 different gRNA. We used the Nucleofector Kit for Primary Mammalian Fibroblasts (VPI-1002). The transfection was performed with 1 × 10^6^ cells per sample that were collected and washed twice in phosphate-buffered saline (PBS) and centrifuged. After that, cells were resuspended in 100μL Nucleofector Solution with 2 μg of Cas9 plasmid, 6 μg of gRNA plasmid and 2 ng GFP plasmid of Amaxa Transfection Kit. The cell and DNA mixture was transferred into a cuvette and electroporated with Amaxa Nucleofector 2B using the Program U-012. After transfection, the cells were cultured in DMEM medium supplemented with 20% fetal bovine serum (FBS) (Sigma), 50μg/mL uridine and 100μg/mL pyruvate for two days and then used for further analysis.

**Fig 3:**
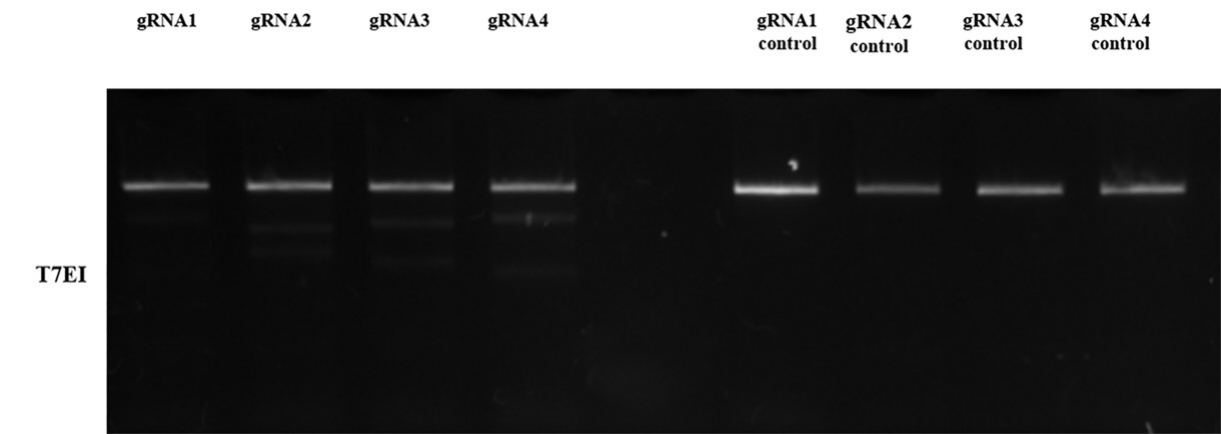
Agarose gel (2.5%) used for the T7EI cleavage assay. Note the different gRNA tested (1 to 4) and controls (1 to 4). The gRNA 1 and 2 with no mutation rate, gRNA 3 with 7.2% mutation rate and gRNA 4 with 10% mutation rate. It’s possible to see 2 brighter bands on gRNA 3 and gRNA 4.

DNA was extracted to perform T7EI gel test to detect the best mutation rate between all 4 gRNA. The mutation rate was quantified by scanning of DNA bands with Image J software (NIH Image-BioLab). gRNA1 and gRNA2 had no mutation. gRNA3 had a 7.2% mutation rate and gRNA4 had a 10% mutation rate. Having chosen the most adequate protocol and gRNA (gRNA4), a new transfection was performed in the same conditions in order to generate edited cells (Fig 3). Two days after transfection, cells were analyzed using the FACSAria flow cytometer and through cellular fluorescence photography.

### Cell cloning through fluorescence activated cell sorting (FACS) of transfected cells

Transfected cells with greater than 70% confluence were isolated by sorting 1 cell/well into 96-well plates using FACS Aria (BD Bioscience) equipped with FACSDiva software for analysis. These cells were cultured in 100μL of DMEM supplemented with FBS, uridine and pyruvate. The cells were incubated at 37°C with 5% CO_2_ and relative humidity at approximately 80% for 20 days.

### T7EI test for quantitating frequencies of indels (insertion or deletion) mutations and Topo Cloning

Genomic DNA was extracted from cells using Qiamp DNA microkit (Qiagen), according to the manufacturer’s protocol. To assess mutation frequencies, T7EI endonuclease assays were performed [15]. The genomic region (gRNA target site) was PCR amplified using primers (Table 1). PCR products were mixed with 2μL NEB buffer 2.0 (New England Biolabs) and water to make a total volume of 20μL. The mixture was denatured and annealed to form heteroduplexes. After that, we performed digestion with 0.32μL T7EI endonuclease (10 units/μL) at 37°C for 30 minutes. To analyze DNA digestion, the products were electrophoresed on a 2.5% agarose, 50% sucrose with proteinase K (20 ng/μL) gel. The mutation rate was quantified by scanning of DNA bands with Image J software (NIH Image-BioLab). The PCR products were sent for Sanger sequencing.

To identify the mutant alleles, the PCR products were cloned by TOPO TA Cloning Kit (Life Technologies) vector prepared according to the manufacturer's instructions and sent for Sanger sequencing.

**Table 1:** Primers sets used for PCR and the T7EI assay.

Name	Primer	Sequence (5'-3’)
TFAM_b1a	Forward	5’GGTGCTCCAAGGTACGAGAA3’
TFAM_b1b	Reverse	5’TAGCCGATTTCCCATAGTGC3’
TFAM_b2a	Forward	5’CAAGGTCGAGGTCGGAATC3’
TFAM_b2b	Reverse	5’GGGCATGATAGTAAATCCGGT3’

### Determination of mtDNA copy number

The mtDNA copy number was estimated [16], samples (fibroblasts in P3) were subjected to total DNA extraction, the DNA was quantified by spectrophotometry (NanoDrop 2000, Thermo Scientific, Waltham, MA, USA) and frozen at -80°C. mtDNA quantification was then performed on a real-time PCR thermocycler (Applied Biosystems, 7500 Fast Real Time PCR System, Foster City, CA, USA) using a commercial assay system (SYBR Green PCR Master Mix; Life Technologies) following the manufacturer instructions. The samples were analyzed in duplicate using the endogenous beta actin gene (ACTB) as a control and primers listed in Table 2.

**Table 2:** Primers used for relative quantification of the target gene (mtDNA) and endogenous control (ACTB).

Target gene (Genbank access)	Primer	Sequence (5'-3’)	Product
ACTB (NM_173979.3)	ACTB-f	5’GGCACCCAGCACAATGAAGA3’	67bp
	ACTB-r	5’GCCAATCCACACGGAGTACTT3’	
MT-RNR 2 (AY526085/ AY126697)	bMT3010-f	5’GCCCTAGAACAGGGCTTAGT3’	87bp
	bMT3096-r	5’GGAGAGGATTTGAATCTCTGG3’	

### Statistical analysis

The statistical analyses were performed using GraphPad Prism 6 (GraphPad Software, Inc. San Diego, CA). One-way Analysis of variance (ANOVA) with p ≤ 0.01, followed by Tukey’s test.

## Results

### Transfection of bovine fibroblasts

**Fig 4:**
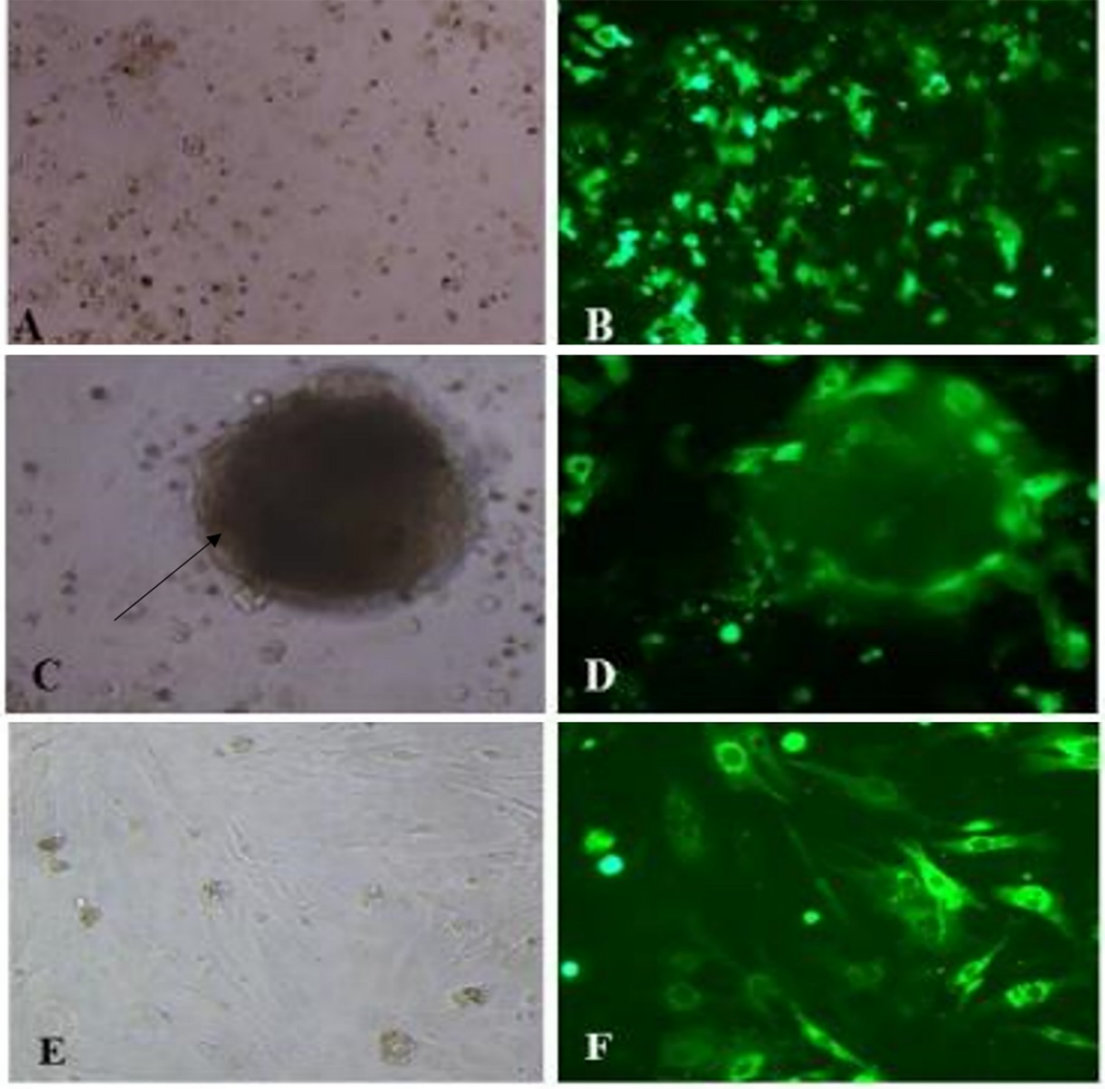
Photomicrographs of fibroblasts after transfection with CRISPR Cas9. In A, C and E note culture of fibroblasts (control). In C note the cluster formed (arrow) and in E adherent cells with 80% confluence. In B, D and F observe the cells positively stained.

After being transfected the cells grew well in culture, showing adherence to plastic and fibroblastoid format and cells positively stained to GFP (Fig 4). We obtained a successful transfection rate of 51.3% GFP positive cells (S1 Fig).

### Cell clone culture

**Fig 5:**
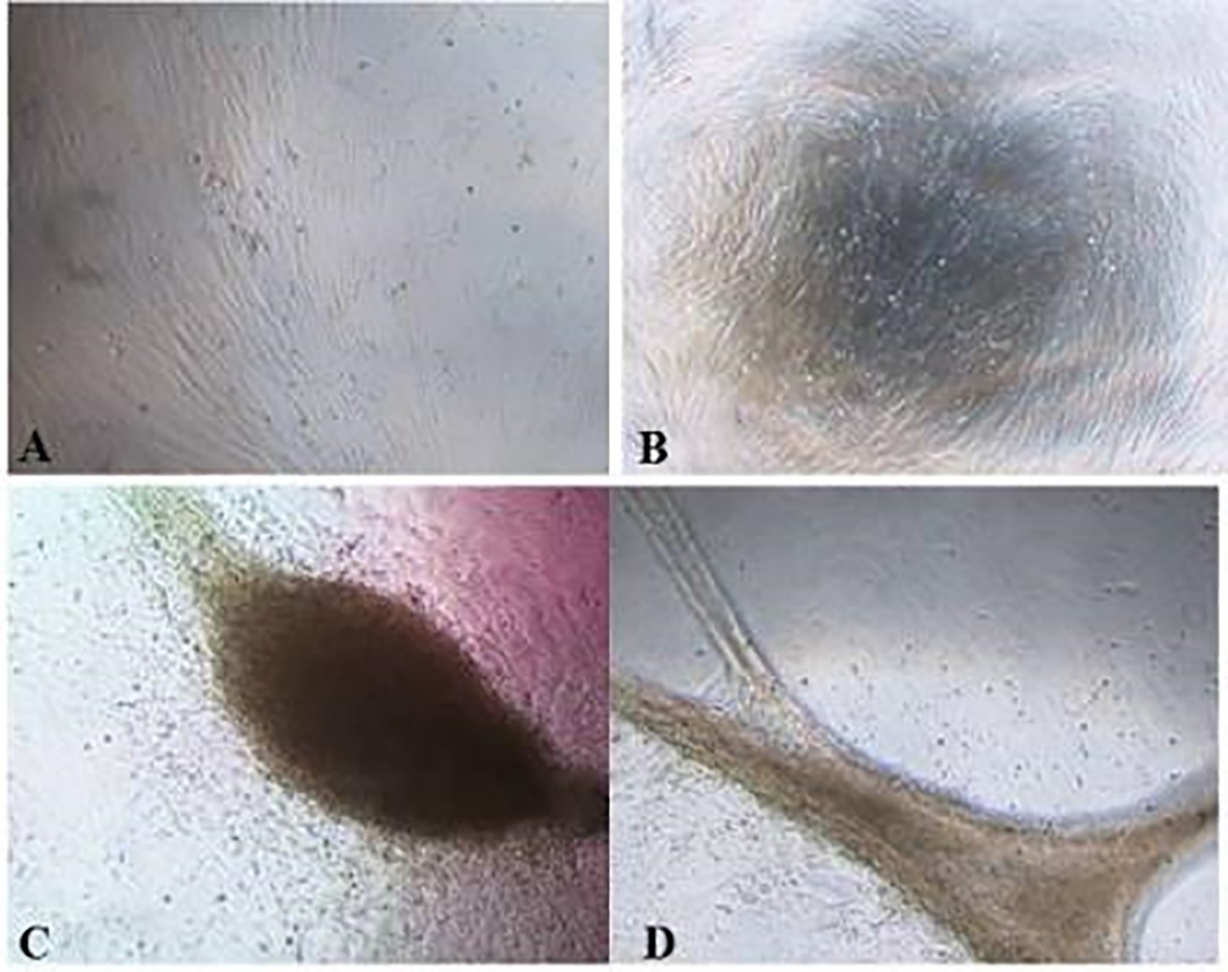
Selected clones. Clones after 7-day growth in 6-well plates. Note adherence to plastic and high cell confluence. In A-B, cells in 96-well plates at 80% confluence; C-D confluent cells in 6-well plates.

The cells were cultured for a period of 20 days, analyzed, and the wells containing individual colonies were selected and then split into 6-well plates, where the cells reached confluence after 4 days in culture. We observed that the clones grew well in culture with uridine and pyruvate showing that the supplementation support the cells in vitro (Fig 5).

### Conventional PCR analysis

PCR was performed for amplification of the target region; for all clones, a band of the expected molecular weight was obtained (see S2 Fig for details).

### T7EI test of clones, TOPO TA Cloning and sequencing

**Fig 6:**
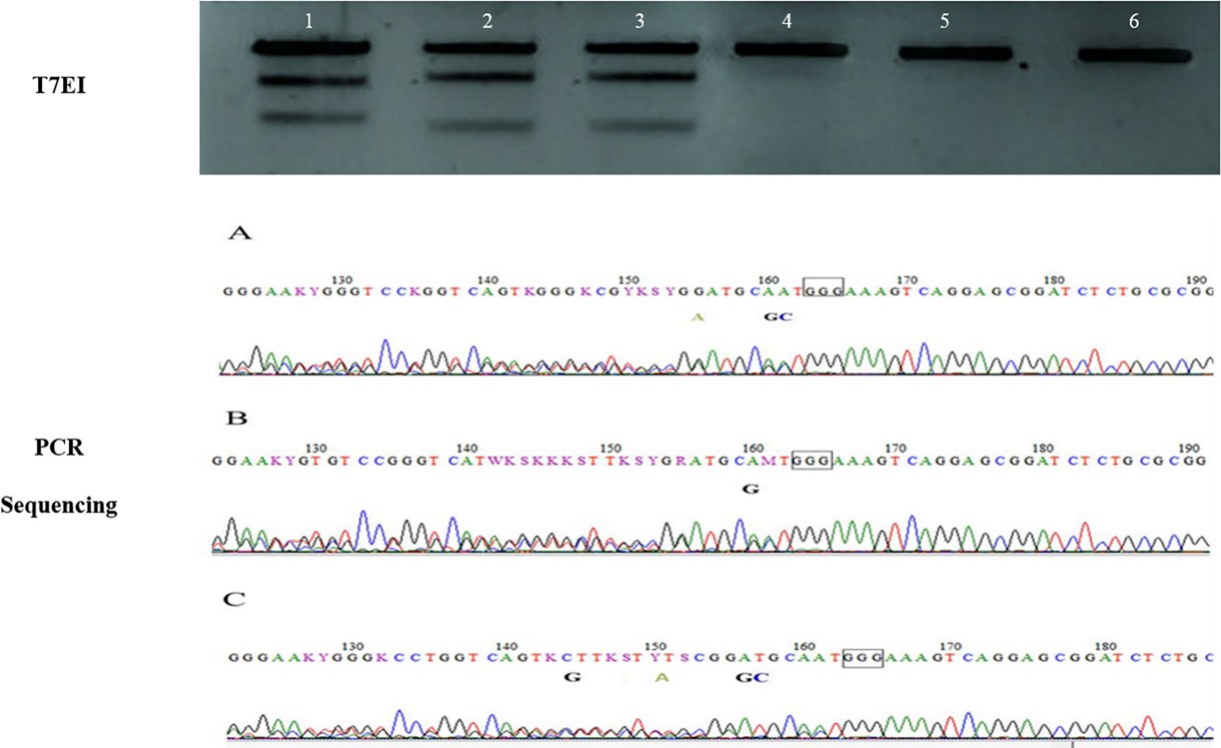
Mutation detection of TFAM gene by T7EI cleavage assay and sequence analysis. In the T7EI assay note mutations in numbers 1 to 3. It’s possible to see 2 bands in the center of the gel and the numbers 4, 5, and 6 are controls of clones 1–3. In the PCR sequencing analysis note A, B and C (nucleotides 163–165) showing the PAM region of the sequence. The cleavage site is 3bp prior to PAM (160–162). In A, note the insertion of two A residues at the site that should be GC. At nucleotide 155, there also occurred an insertion of G, normally an A. In B, note the insertion of an A substitution for G. In C the insertion of AT nucleotides involving positions 156 and 157. This site is normally GC. Also insertion of a T at the A site of nucleotide 151, and insertion of C at the G site at nucleotide 144.

**Fig 7:**
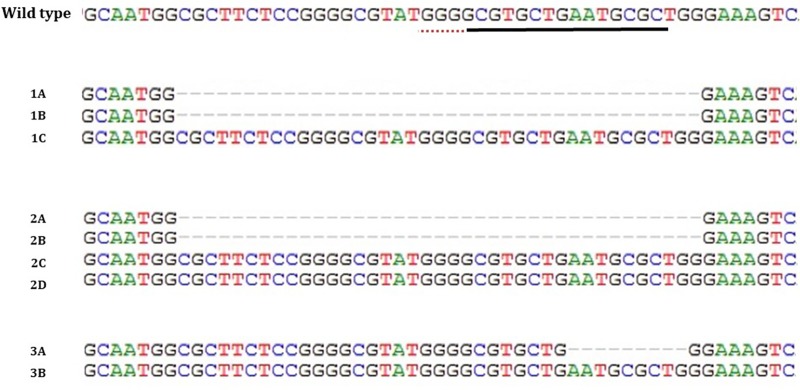
Mutation detection of TFAM. Sequences of alleles identified by Sanger sequencing. The sequence of gRNA is shown in horizontal black underlined region, the PAM site is the horizontal red underline. In 1A, 1B, 2A, 2B sequenced colonies mutations with 39bp deletion (dotted); 1C, 2C, 2D, 3B sequenced colonies wild-type alleles. In 3A sequenced colonies mutations with 9bp deletion (dotted).

After T7EI tests, we observed that 7 clones presented mutations. Through DNA sequencing, we were able to confirm the mutations. Close to the target region, the presence of 2 peaks was noticed, probably due to heterozygous mutations. The CRISPR mechanism cleaves 3 base pairs prior to the PAM (Protospacer adjacent motif) region and in all of our clones, we noticed a deletion in this region (Fig 6). We confirmed the heterozygous mutations through PCR products that were cloned by TOPO TA Cloning Kit (Life Technologies) vector and sent for Sanger sequencing. Several colonies from the same clone were analyzed. The sequencing results showed in all of the samples only one allele edited (Fig 7).

### Determination of mtDNA copy number

**Fig 8:**
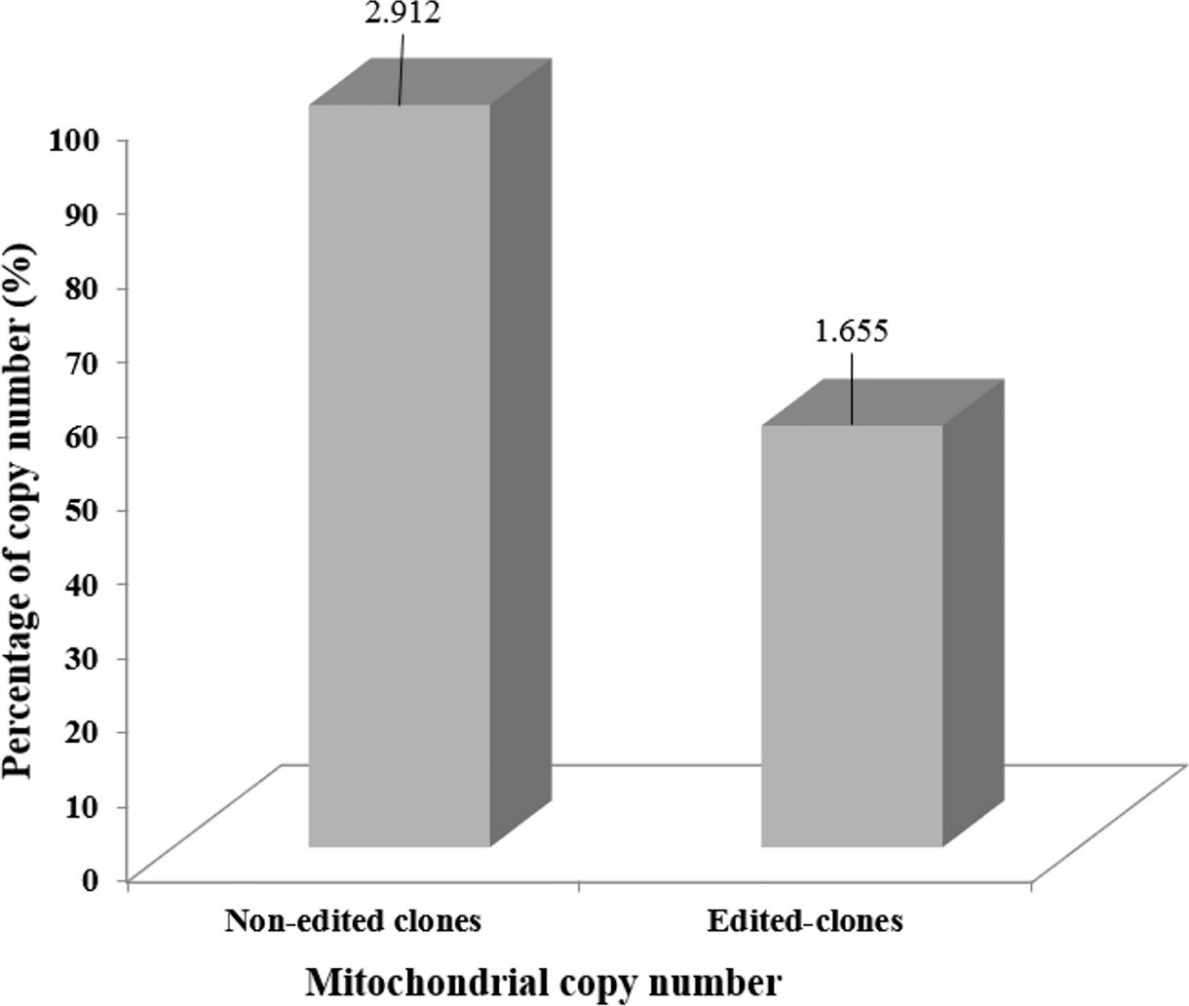
Number of copies of mtDNA per cell, non-edited clones (control) and edited clones. Note the 100% percentage of copy number to non-edited clones (used as reference) with 2.912 mitochondrial number copy and 56.8% to edited-clones with 1.655 mitochondrial number copies. Significative difference (P ≤ 0.01).

Regarding the determination of the mtDNA copy number we observed a decrease in the copy number in the edited clones (heterozygous) when compared to non-edited (control) clones of bovine fibroblasts. The non-edited clones showed 2.912 copy number on average and the edited clones 1.655 mtDNA copy number (Fig 8). These results reveal that the CRISPR/Cas9 editing was efficient and even though only one allele was edited it was enough to present a significative difference (P ≤ 0.01) in the mtDNA copy number.

## Discussion

Genetic editing in cattle is an important tool for generating gene knockouts in animal models, such as in herds for pharmaceutical purposes. These genetic modifications are of extreme importance for both agricultural science and biomedical applications, rendering this particular animal model more suitable for gene therapy when compared to laboratory rodent models [5,17–19].

CRISPR/Cas9 has already been used in bovids and has demonstrated the feasibility of manipulation of the Nanog gene. This mechanism was highly efficient in both bovine embryos and pluripotent stem cells [20].

In our study, we used CRISPR/Cas9 technology to edit the TFAM gene in bovine fibroblasts. We designed a gRNA using the website crispr.dbcls.jp, following the protocol previously reported for zebrafish [21]. For transfection of bovine fibroblasts, we used AMAXA Nucleofector 2B equipment because it is a method based on a combination of solutions and electrical parameters that directly transfers DNA to cell nuclei. This technique has been used by several authors in many primary cell types [22–24].

Many authors report the use of electroporation in bovine fibroblasts and found it to be very efficient for DNA transfection, as well as for fibroblastoid cells of other mammals [25].

In our study, we performed GFP plasmid transfection into bovine fibroblasts. As a result of our experiments to generate DNA editing using CRISPR/Cas9, when pGFP plasmid was combined with gRNA and Cas9 expression plasmids, we observed a transfection rate of 51.8% using Amaxa running program U-012.

As the disruption of the TFAM gene is expected to be lethal, in order to maintain the post-transfected cells in culture we supplemented the culture medium with uridine and pyruvate, similar to Rho 0 cells that are capable of surviving without mtDNA when exposed to these conditions. Rho 0 cells are entirely dependent on glycolysis for their energy demands, as well as being auxotrophic for uridine because the enzyme responsible for the synthesis of this nucleoside (dehydrogenase dihydroorotate) is located in the inner mitochondrial membrane and requires the function of the electron transport chain for its activity [26–27].

Rho 0 cells require a medium supplemented with uridine and pyruvate as energy sources so they can proliferate and survive [26–28]. Thus, transfected cells treated with uridine and pyruvate have the ability to be maintained in vitro similar to cells that do contain mtDNA. To carry out supplementation, we followed the same protocol as used in bovine fetal fibroblasts treated with ethidium bromide to evaluate the effects on the number of mitochondrial DNA copies and their actions involving cellular metabolism. The treated and control fibroblast cultures supplemented with uridine and pyruvate were stably maintained in culture, and it was observed that such treatment did not affect cellular growth rate and that supplementation was able to support normal cell proliferation [29–30]. This is similar to our findings, in that cells remained viable post-transfection, without affecting cell growth rate.

In the CCR5 gene, gene knockout was performed in fibroblasts and in human embryonic stem cells through transfection using the AMAXA protocol, with a mutation rate of 19% in fibroblasts and 23% in H9 embryonic stem cells from humans [31].

In sheep fibroblasts, knockout was performed in the myostatin gene, and by PCR assays, a cleavage efficiency of 19.3% was identified [32].

In our findings, the initial mutation rate identified in bovine fibroblasts was 7.2% and 10%, but we found that increasing the concentration of Cas9 and gRNA could raise the rate to 40% efficiency. However, when clones from cells transfected with the highest amount of Cas9 plasmid were cultured, we noticed that they did not grow well and soon underwent apoptosis, suggesting that high concentrations of Cas9 may be toxic to cells. Higher concentrations of Cas9 result in higher mutation rates at target sites, but may possibly generate off-target modifications, which may contribute to toxicity [33].

After refining our methods we generated a mutation in fibroblasts; with the analysis of the clones we found mutations and we isolated heterozygous clones carrying one mutant and one wild-type TFAM allele. Therefore, we believe that we generated a new interesting model for further study because heterozygosity should decrease the amount of TFAM protein by 50%, possibly leading to mitochondrial dysfunctions and the generation of diseases, as overexpression (due to a high mtDNA compaction, thus hindering replication) or low expression can lead to decreased numbers of mitochondrial copies [34].

Research in mice with heterozygous TFAM mutation showed a reduction in mtDNA copy number and lethality in TFAM homozygosis [35].

The heterozygous TFAM -/+ mouse had decreased copy numbers of myocardial mtDNA and homozygous -/- mice showed depletion of mtDNA with a decrease in OXPHOS and death during embryonic development [36]. Conversely, high TFAM expression in transgenic mice increased the number of mtDNA copies, showing that this increase may improve severe symptoms of certain mitochondrial diseases [37]. It has also been reported that an unbalance in the mtDNA copy number due to TFAM alterations may be associated with many neurodegenerative and cardiac diseases, as well as cancer [38–39].

Other studies show that heterozygous TFAM make mice more prone to suffer metastasis in an intestinal cancer model [40]

Regarding the cardiac issue, recent studies have demonstrated that TFAM inhibits the NFAT4-MMP9 proteolytic pathway in TFAM transgenic mice subjected to aorticbanding-induced heart failure, reducing pathological cardiac remodeling like hypertrophy and other HF associated factors. When KO’d, the absence of TFAM induces the activation of proteases Calpain1, MMP9 and NFAT4, resulting in pathological remodeling of the heart and increased ROS production. So, when overexpressed, TFAM reduced all of these HF effects by inhibiting the NFAT4-MMP9 cardiac remodeling cascade, postulating TFAM as a possible therapeutic approach to cardiac pathologies [38–39]

Other studies addressed the relation between Krüppel-like (KLF) transcription factors, TFAM and cancer. KLF are DNA-binding proteins that regulate gene expression and the noted research showed that they play a key role in the differentiation and development of tumors and the regulation of carcinogenesis, being capable of both promoting and suppressing tumor appearance, according to the cellular context. In this research the quantities of KLF16 were altered showing that it helps to modulate TFAM in glioma cells, hindering (or otherwise increasing) the cancerous cells proliferation rate by targeting the main mitochondrial transcription factor, thus demonstrating a new potential approach for the TFAM gene [41].

Considering the mtDNA copy number our results were consistent with the literature because our edited fibroblasts -/+ also showed the expected decrease in mtDNA when compared to unedited clones, confirming the important role of TFAM in mtDNA modulation and preservation. From our studies, the CRISPR/Cas9 design was efficient to generate clones with mutation through disruption of *TFAM* gene. This new cell line with heterozygosity of the *TFAM* gene affords a tool to confirm that TFAM has direct action involving mtDNA in bovids. For example, it can play a fundamental role in the maintenance of genetic stability, inheritance and segregation.

## Supporting information

S1 Fig Diagram of flow cytometry. Note the 51.3% of GFP positive cells.
(JPG)

S2 Fig Conventional PCR analysis and 1.5% agarose gel electrophoresis. PCR of clones (1 to 7) showing the 361 bp amplified region.
(TIF)
