# Supplementary figures and images for: Edition of TFAM gene by CRISPR/Cas9 technology in bovine model
Source: PLoS One. 2019 Mar 7;14(3):e0213376. doi: 10.1371/journal.pone.0213376 (PMC6405117; doi:10.1371/journal.pone.0213376)

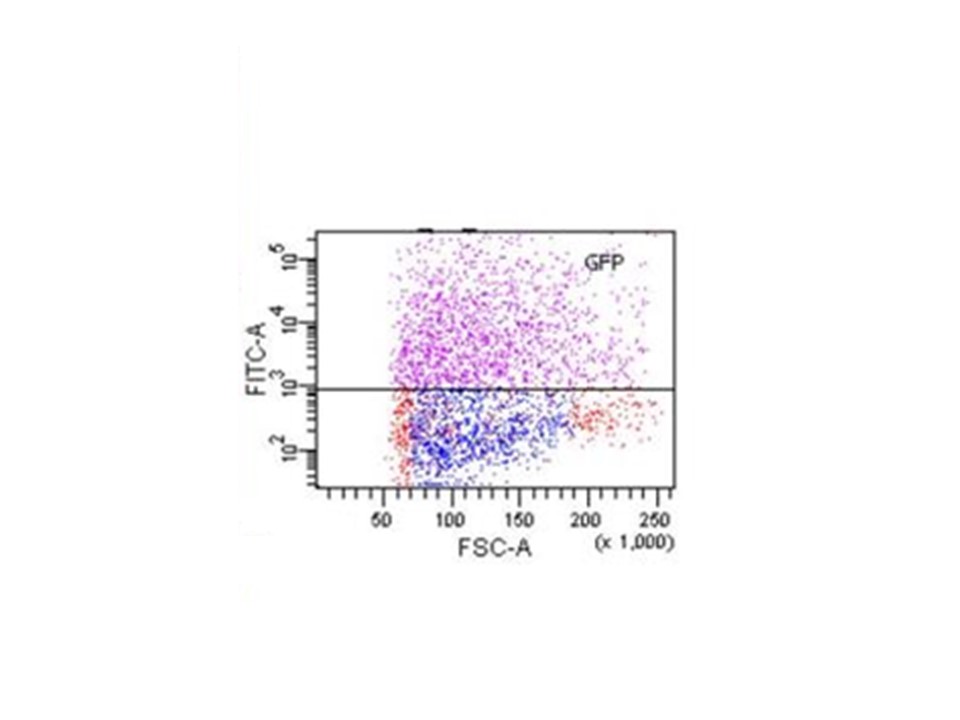

Supplement: S1 Fig — Note the 51.3% of GFP positive cells. (JPG) [file pone.0213376.s001.jpg]

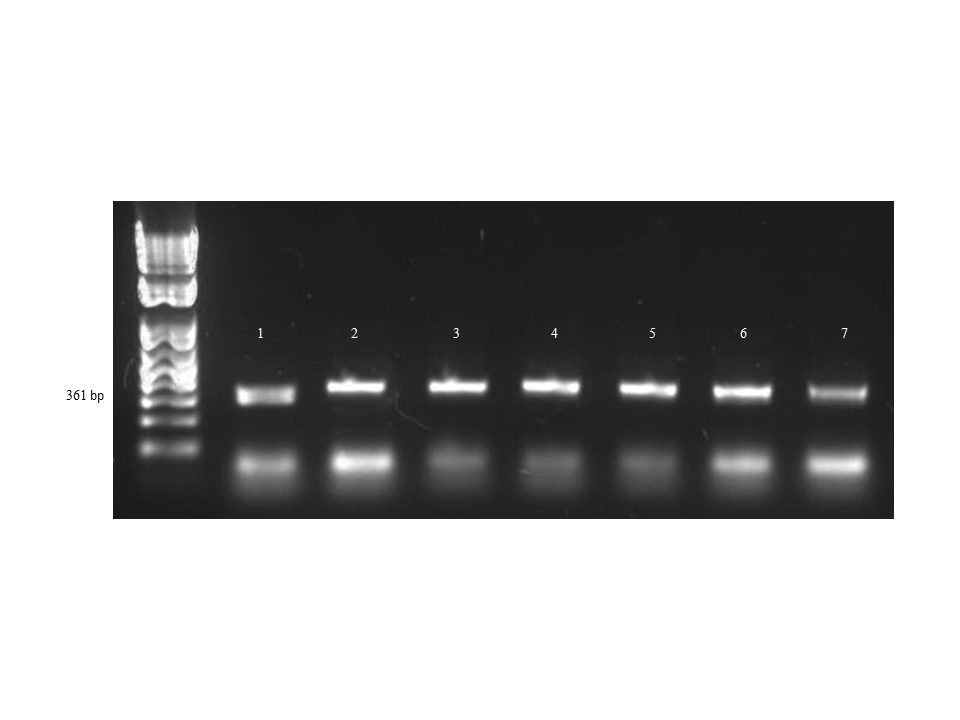

Supplement: S2 Fig — PCR of clones (1 to 7) showing the 361 bp amplified region. (TIF) [file pone.0213376.s002.tif]
